# Depression levels in patients with hyperemesis gravidarum: a prospective case–control study

**DOI:** 10.1186/s40064-015-0820-2

**Published:** 2015-01-24

**Authors:** Hüseyin Aksoy, Ülkü Aksoy, Özge İdem Karadağ, Yunus Hacimusalar, Gökhan Açmaz, Gülsüm Aykut, Fulya Çağlı, Burak Yücel, Turgut Aydın, Mustafa Alparslan Babayiğit

**Affiliations:** Department of Obstetrics and Gynecology, Kayseri Military Hospital, Kayseri, Turkey; Department of Obstetrics and Gynecology, Kayseri Memorial Hospital, Kayseri, Turkey; Department of Obstetrics and Gynecology, Kayseri Acıbadem Hospital, Kayseri, Turkey; Department of Psychiatry, Kayseri Education and Research Hospital of Medicine, Kayseri, Turkey; Department of Obstetrics and Gynecology, Kayseri Education and Research Hospital of Medicine, Kayseri, Turkey; Department of Public Health and Epidemiology, Gülhane Military Faculty of Medicine, Ankara, Turkey

**Keywords:** Depression, Hyperemesis, Pregnancy

## Abstract

Hyperemesis gravidarum (HG) is a condition characterized by severe, intractable nausea and vomiting in early pregnancy. It affects about 0.3–2% of all pregnancies and is thought that HG is a multifactorial disease resulting from the combination of various unrelated conditions such as genetic, hormonal and psychiatric. Although there are studies investigating the relationship between anxiety, depression and HG; however, none have sufficiently clarified this link. The aim of this prospective case–control study was to investigate the possible relationship between depression and HG and compare the prevalence of depression disorders in pregnant women with and without HG.

A prospective case–control study was performed at our tertiary referral centre between December 2013 and July 2014. The study group consisted of 78 pregnant women with HG and the control group consisted of 82 healthy pregnant women who never had experienced any nausea and vomiting. No study participants had any pre-pregnancy history of any psychiatric disorder including depression. Structured Clinical Interview for Diagnostic (SCID-I) and Statistical Manual of Mental Disorders Fourth Edition (DSM-IV) was used to evaluate symptoms of depression. Beck Depression Inventory (BDI) was administered to patients during the psychiatric interview and was evaluated by the same psychiatrist.

The mean BDI scores in HG study and healthy control groups were 18.97 ± 9.85 and 6.36 ± 5.61, respectively (p < 0.001). Among the 78 women in the HG study population, 42 (53.9%) of patients had moderate or severe depression disorder. Only 6.1% of patients in the control group had moderate or severe depression.

In conclusion, the findings of this study indicated that psychological distress associated with HG was a direct consequence rather than a cause of HG. Therefore, patients with HG during pregnancy should be evaluated with respect to mood disorders as much as their medical conditions.

## Background

The majority of pregnant women experience varying degrees of severity from mild to severe symptoms of nausea and vomiting during pregnancy (NVP) known as morning sickness. NVP affects about 70–80 percent of pregnant women (Gadsby et al. 1993,Gazmararian et al. 2002). Hyperemesis gravidarum (HG) is an extreme form of morning sickness during early pregnancy. HG is a condition characterized by severe, intractable nausea and vomiting in early pregnancy and associated with dehydration, ketonuria, fluid- electrolyte imbalance, nutrition deficiency and weight loss (Verberg et al. 2005,Fairweather 1968). HG is one of the most common pregnancy-related diseases, and is a leading cause of maternal hospitalization during pregnancy (Gazmararian et al. 2002, Ismail and Kenny 2007). It affects about 0.3–2% of all pregnancies and is more prevalent when coexisting conditions such as trophoblastic disease, multiple pregnancies and other conditions associated with high levels of human chorionic gonadotropin (hCG) (Verberg et al. 2005, Ismail and Kenny 2007). Although the exact etiology and pathophysiology of HG is not completely known, it is currently accepted that HG is a multifactorial disorder of pregnancy. There are several theories regarding the etiology and pathophysiology of HG, but the exact cause and mechanism remain controversial. It is thought that HG is a multifactorial disease resulting from the combination of various unrelated conditions such as genetic, environmental, hormonal and psychiatric (Verberg et al. 2005, Uguz et al. 2012, Tan et al. 2010, Hendler et al. 2004, Fejzo and Macgibbon 2012, Vikanes et al. 2010). The physiological basis of HG is often reported, most consistently with hormonal changes such as high levels of human chorionic gonadotropin, increased estrogen, progesterone and thyroid hormone levels (Verberg et al. 2005, Ismail and Kenny 2007). Some other pathologic factors, such as gastrointestinal dysfunction, hepatic abnormalities, lipid alterations, overactivation of sympathetic nervous system and infection, in addition to endocrine factors of hyperemesis gravidarum may play a role in etiology and pathophysiology of this medical condition (Verberg et al. 2005, Koch 2002, Ustün et al. 2004, Lee et al. 2005, Niemeijer et al. 2014). Along with all of these physiological changes, psychosomatic factors may also play a role in this complex and multifactorial condition.

Despite the common psychosomatic symptoms observed in patients with HG, the psychological components of the disease have not been fully understood. The psychological basis of illness is controversial. Many studies have investigated the association between HG and maternal psychological morbidity; however, most studies have provided conflicting results (Swallow et al. 2004, Köken et al. 2008, Seng et al. 2007, Pirimoglu et al. 2010). The relationship between anxiety, depression and HG has been also investigated in some other studies previously, and these studies have also provided conflicting results (Bozzo et al. 2011, Jahangiri et al. 2011, Kramer et al. 2013, Chou et al. 2003, Bozzo et al. 2006). In addition to conflicting results of studies, most of the studies on psychological components of illness had significant limitations such as retrospective study design, lack of proper sample size, lack of control group, lack of objective diagnosis criteria, bias and variable definitions of disease (Bailit 2005, Dodds et al. 2006, Fejzo et al. 2009, Kitamura et al. 1996). A possible relation between anxiety, depression and HG has been reported by some authors; while others have not found this connection (Uguz et al. 2012, Swallow et al. 2004, Köken et al. 2008, Bozzo et al. 2011, Jahangiri et al. 2011, Chou et al. 2003, Bozzo et al. 2006).

The aim of this prospective case–control study was to compare the prevalence of depression disorders that first occur during pregnancy in women with HG compared to healthy pregnant controls.

## Results

A total of 198 patients who admitted to our obstetric department for hospitalization due to HG and routine antenatal care were evaluated for eligibility. Thirty-eight patients were excluded, of which 21 refused to participate in the study and 17 did not meet the inclusion criteria. The final study group was composed of 160 subjects.

The mean age was 27.21 ± 5.91 years in HG study group and 26.19 ± 5.46 years was in the healthy control group. The study groups did not differ with respect to mean age, gravidity, parity, gestational age and body mass index (BMI). Some demographic and clinical characteristics of patients for each of the groups are illustrated in Table 1. All of the participants were married and were in the first trimester of gestation.

**Table 1:** **Some demographic and clinical characteristics of groups**

	**HG Study group**	**Healthy control group**	***p***
	**(n = 78)**	**(n = 82)**	
**Age**	25.19 ± 5.39	26.56 ± 6.31	0.397*
**BMI**	24,81 ± 6,97	25,06 ± 7,79	0,675*
**Gravidity**	2.46 ± 1.20	2.10 ± 1.07	0.053**
**Parity**	1.26 ± 0.97	0.98 ± 0.90	0.066**
**Gestational age**	8.51 ± 2.42	8.36 ± 2.50	0.672**

*Student’s *T* Test.

**Mann Whitney *U* Test.

The mean BDI scores HG study and healthy control groups were 18.97 ± 9.85 and 6.36 ± 5.61, respectively. A significant difference was found between two groups regarding mean BDI scores (p < 0.001). Mean BDI scores of the participants in two groups are presented in Table 2.

**Table 2:** **The comparison between mean BDI scores of The HG and control groups**

	**HG Study group**	**Healthy control group**	***p***
	**(n = 78)**	**(n = 82)**	
**Mean BDI Score**	18.97 ± 9.85	6.36 ± 5.61	<0.001*

*Student’s *T* Test.

The prevalence of depression disorder of patients in each group according to depression degree are shown in Table 3.

**Table 3:** **The prevalence of depression disorders in groups**

**Psychiatric disorders**	**HG Study group**	**Healthy control group**	***p***
	**n (%)**	**n (%)**	
**Depression**			
**No**	10 (12.8)	58 (70.7)	<0.001*
**Mild**	26 (33.3)	19 (23.2)	<0.001*
**Moderate**	30 38.5)	4 (4.9)	<0.001*
**Severe**	12 (15.4)	1 (1.2)	<0.001*

*Chi-square Test.

Among the 78 pregnant women with HG, 10 patients (12.8%) with the BDI score 0–9 had no depression, 12 patients (15.4%) with the BDI score 10–16 had mild depression, 30 patients (38.5%) with the BDI score 17–29 had moderate depression and 12 patients (15.4%) with the BDI score 30–63 suffered from severe depression. In healthy control group, 58 (70.7%) of patients had no depression, 19 (23.2%) mild, 4 (4.9%) moderate, and 1 (1.2%) had severe depression. When the results were compared to the control group, significantly higher mean BDI scores were found in the HG group (p < 0.001).

## Discussion

Hyperemesis gravidarum is an extreme form of nausea and vomiting in early pregnancy and is a leading cause of maternal hospitalization during pregnancy. It affects approximately 0.3–2% of all pregnancies (Gazmararian et al. 2002, Verberg et al. 2005, Ismail and Kenny 2007). In this study, we investigated the possible relationship between depression and HG and compared the prevalence of depression disorder in patients with and without HG. The most important finding of this study is that more than half of the patients with HG had moderate or severe depression disorder, and these prevalence rates were higher than the rates for healthy control subjects.

In the literature, there are many studies investigating the relationship between anxiety, depression and HG, but prospective case-controlled studies specific to anxiety and depression disorders in patients with HG are limited (Tan et al. 2010, Swallow et al. 2004, Köken et al. 2008, Seng et al. 2007, Pirimoglu et al. 2010, Bashiri et al. 1995). However, most studies investigating this link have provided conflicting results (Swallow et al. 2004, Bozzo et al. 2011, Jahangiri et al. 2011, Kramer et al. 2013, Köken et al. 2008, Chou et al. 2003, Bozzo et al. 2006). Several studies have also demonstrated that there is a possible relationship between anxiety, depression and HG (Uguz et al. 2012, Tan et al. 2010). Although there is a potential relationship between psychiatric disorders and HG during pregnancy, the psychological dimension of the disease is poorly understood. In 2008, Tan et al. analyzed 209 hospitalized patients with HG to determine the prevalence and the risk factors of anxiety and depression at their first hospitalization for HG. They reported that anxiety and depression disorders were common in women affected by HG and the psychological distress associated with HG was a direct consequence rather than a cause of HG (Tan et al. 2010). The findings of our study were consistent with Tan’s study; since no patients included in our study had depression prior to their pregnancies. Köken et al. performed a prospective analysis of 230 women with NVP and a significant correlation between severity of NVP and both anxiety and depression scores were found (Köken et al. 2008). However, the Hospital Anxiety and Depression Scale was used in these studies as the psychiatric symptom scale, and this scale had low clinical value. The potential relationship between psychiatric disorders and HG during pregnancy was described by some other researchers (Swallow et al. 2004, Chou et al. 2003, Annagür et al. 2014). These studies using psychiatric symptom scales suggested that nausea and vomiting in early pregnancy were significantly correlated with depression symptoms. In an extensive study conducted by, Seng et al. performed a retrospective analysis of 11,016 singelton pregnancies over a 4-year period (Seng et al. 2007). The authors analyzed insurance data for all 11,016 patients, 208 of whom had HG. It was found that patients with HG had more frequent psychiatric diagnosis preceding the pregnancy compared to the control subjects. In another study conducted by Pirimoglu et al. using a psychiatric symptom scales showed that women with HG had higher psychological distress scores than those in the control group. In this prospective case–control study, data of 34 hospitalized women with HG were analyzed over a 2-year period (Pirimoglu et al. 2010). In a very recent article, Budzyński et al. analyzed changes in brain-gut axis in the pathogenesis of Helicobacter pylori infection (Budzyński and Kłopocka 2014). They concluded that the bidirectional relationship between H. pylori infection and the brain-gut axis influenced both the contagion process and the host’s neuroendocrine-immunological reaction which resulted in alterations in food intake, appetite and cognitive functions. Unlike this study, we did not evaluate the participants for H.pylori infection.

In our study, the current prevalence rate of moderate-severe depression disorder in patients with HG was 53.9%. Epidemiological studies have indicated that 2.2%–15.6% of women in the first trimester and 4.7%–17.3% of women in the general population have anxiety or depression disorder (Bennett et al. 2004, Farias et al. 2013, Bödecs et al. 2013, Gavin et al. 2005, Sartorius et al. 1996, Regier et al. 1993, de Girolamo et al. 2006, Kessler et al. 1994). Comparing our results to those from large-scale epidemiological studies, our study suggests that patients with HG more commonly experience depression disorders than healthy pregnant women without HG or women in the general population. In a systematic review, Gavin et al. investigated the prevalence and incidence of perinatal depression (Gavin et al. 2005). Authors reported that the prevalence of depression during pregnancy was approximately 8–12%. The results of this systematic review are similar to our control group’s findings**.** In our study, only 6.1% of patients in the healthy pregnant group had moderate or severe depression. The mean BDI score in HG study group was 18.97 ± 9.85. Importantly, significant difference with regard to mean BDI score was found between HG and healthy control groups. These findings were comparable with mean BDI score of 15.38 ± 9.18 for the patients with HG in the first trimester reported in a previous study conducted by Annagür (Annagür et al. 2013). Annagür et al. found that the rate of mood disorder was 14.9% in their study samples. Another study conducted by the same author Annagür et al., similar BDI scores were reported. The mean BDI score for the patients with HG was 15.7 ± 9.3.

Our study findings were inconsistent with some of the few studies on this subject (Bozzo et al. 2011, Jahangiri et al. 2011, Kramer et al. 2013). In contrast to our data, Bozzo et al. and Jahangiri et al. demonstrated that there was no association between depressive symptoms and NVP. Bozzo et al. evaluated the relationship between depressive symptoms and NVP in a group of 57 singleton pregnancies over all pregnancy and a 18-week postpartum period. In the study conducted by Jahangiri et al., data of 45 singleton and 12 twin gestations were analyzed. This discrepancy in findings may be related to differences in sample size and gestational age because the incidence and severity of nausea and vomiting in HG varies according to the gestational week. The main reason for discrepancy in findings may be related to differences in the psychiatric symptom scales and questionnaires used in studies. It may also be related to the differences in the study protocols and the possible biases in the previous studies.

There are some limitations to our study. The data collection from the only one obstetrics clinic is the potential limitation of the study. The absence of longitudinal data as well as control data on psychological symptoms after recovery from illness in the following gestational weeks is another limitation of this study. In this current study, psychological distress scores were obtained in only physical illness period in the first trimester of gestation. The impact of the paper would have been better if we could make a comparison between healty pregnant women, patients with HG and women being admitted to the hospital with similar symptoms such as nausea and vomiting with the flu or nausea and vomiting due to other illnesses. Another weak point is that the BDI has questions that may be directly related to nausea and vomiting rather than a depression disorder including “loss of pleasure, loss of energy, changes in sleep pattern, changes in appetite, tiredness or fatigue, etc.”

This study has many strengths. The major strength of this study was its prospective nature and the inclusion of a control group of healthy pregnant women without HG. Other important strengths of the study include wide and strict exclusion criteria, and the use of both psychiatric interviews and self-report measures standardized for study population to assess anxiety and depression scores. In addition, all psychiatric interviews were performed by a single experienced psychiatrist (YU) to avoid possible observer-dependent factors.

HG appears to occur as a complex interaction of biological, psychological, and sociocultural factors. Physiological changes associated with pregnancy interact with each woman’s psychologic state. Along with all of these physiological changes, psychological factors are also suspected in the etiology and pathogenesis of HG, but the causal relationship between HG and depression disorder is unclear.

## Conclusions

Depression disorder is common in patients with HG. The findings of this study indicated that psychological distress associated with HG was a direct consequence rather than a cause of HG. Therefore, patients with HG during pregnancy should be evaluated with respect to mood disorders as much as their medical conditions. Psychiatric counseling may be helpful in patients with HG to assess the anxiety and depression degree and provide optimal management, care and support for these patients. However, further more larger scale, prospective controlled and homogeneous studies are needed to confirm these results.

## Methods

A prospective case–control study was performed at our Obstetrics and Gynecology Clinic of Kayseri Education and Research Hospital of Medicine, a tertiary referral centre in Turkey, between December 2013 and July 2014. The study was approved by the institutional ethics committee and all participants signed an informed consent form regarding to participate in this study.

The study group consisted of 78 pregnant women with HG who were hospitalized in our Obstetric Inpatient Clinic and 82 healthy pregnant women who admitted to our obstetric outpatient clinic for routine antenatal care without nausea and vomiting constituted our healthy control group. All of the patients were matched for age, parity, body mass index and gestational age. All patients included in the study had a singleton pregnancy.

Inclusion criteria for this study were: age 18 years or older; a single viable intrauterine pregnancy confirmed by precise date of the last menstrual period and an ultrasound scan; written approval and willingness to comply with the study. Our exclusion criteria for all groups were as follows: history of any medical problem (e.g., endocrine abnormalities, gastrointestinal, cardiovascular and pulmonary system diseases) or psychiatric disorder (e.g., depression, anxiety, bipolar disorder, delirium, eating disorders, and psychotic disorder), multiple pregnancies, known obstetric complications such as imminent abortion, gestational trophoblastic disease and ectopic pregnancy, any systemic diseases or medication (including antidepressant, anti-psychotic or other psychiatric drugs during the last 6 months) that would affect the test results, current or past history of illegal drug or narcotic use and cognitive incompetence which can make difficult to understand how to score The Beck Depression Inventory. No study participants had any pre-pregnancy history of any psychiatric disorder including depression. Patients with recurrent admissions for HG to our Obstetric Inpatient Clinic were only recruited during their first hospitalization.

Diagnosis of HG was made based on the clinical criteria (observation of at least three vomiting episodes a day, loss of at least 5% of total body weight and ketonuria) and other causes of vomiting, such as gastroenteritis, cholecystitis, acute pancreatitis, gastric outlet obstruction, pyelonephritis, primary hyperthyroidism, primary hyperparathyroidism, or liver dysfunction were excluded. All patients who met eligibility criteria were sequentially recruited by research coordinator (U.A.) at the study site. All participants were informed about the study and gave their informed written consents for study participation. After informed consent, the participant completed an enrollment questionnaire assessing sociodemographic characteristics and medical information. The final study group was composed of 160 subjects. Each subject underwent a comprehensive medical and obstetric examination along with obstetric ultrasound to confirm the intrauterine pregnancy as well as to exclude any relevant obstetric pathology (e.g., twin pregnancy, molar pregnancy or missed abortion). Gestational age was determined with ultrasound screening on the basis of the last menstrual period. All obstetric procedures (routine antenatal obstetric sonography, laboratory tests, treatments of patients) and study informing were performed by a single obstetrician (U.A.) to avoid interobserver variability. After recording the socio-demographic and clinical characteristics of participants in the obstetric clinic, patients were referred to the psychiatry department. All of the psychiatric interviews were conducted by a single experienced psychiatrist (Y.H.) to avoid possible observer-dependent factors (counseling, patient preparation, moral and psychological support). Structured Clinical Interview for Diagnostic and Statistical Manual of Mental Disorders, Fourth Edition (DSM-IV) (SCID-I), was used to evaluate symptoms of depression. BDI was administered to patients during the psychiatric interview and were evaluated by the same psychiatrist.

### Measures

SCID-I is a semi-structured interview instrument used to establish Axis I psychiatric disorders according to DSM-IV criteria (First et al. 1997). This instrument is widely used in clinical practice and for research purposes all over the world. The interview instruments have been standardized for Turkish populations (Özkürkçügil et al. 1999). In this study, we used The Beck Depression Inventory.

#### BDI

The BDI is a 21-item multiple-choice self-report inventory that measures the severity of depression with a 0–3 scoring system (0 = “least” and 3 = “most”) (Beck et al. 1988). The total score is obtained by the sum of all BDI item scores. The total score ranges 0–63 with higher scores indicating more severe depressive symptoms. The Turkish version of the BDI used in this study has been validated in Turkish populations (Hisli 1989). Scores from 0 to 9 represent no depression, scores of 10 to 16 indicate mild depression, scores of 17 to 29 indicate moderate depression, and scores of 30 to 63 indicate severe depression (Aydemir and Köroglu 2000). Questionnaire takes approximately 15 minutes to fill. However, this period may vary depending on the patient’s level of education.

### Statistical analysis

Continuous variables were expressed as mean ± standard deviation and median (minimum - maximum), whereas categorical variables were denoted as numbers or percentages where appropriate. Kolmogorov-Smirnov Goodness of Fit test was used to test the distribution of data. When the distribution was normal, Student’s *T* Test was used, otherwise analysis were done by Mann Whitney *U* Test. Chi-square Test was used for the comparison of the categorical data. Collected data were analyzed by Statistical Package for Social Sciences version 15.0 (SPSS Inc., Chicago, IL, USA). Two-tailed p value less than 0.05 was accepted to be statistically significant.

## Abbreviations

NVP: Nausea and vomiting during pregnancy
HG: Hyperemesis gravidarum
hCG: Human chorionic gonadotropin
BMI: Body mass index
BDI: Beck depression inventory
DSM-IV: Diagnostic and ststistical manuel of mental disorders
SCID-I: Structured clinical interview for DSM disorders
